# Fabrication and validation of flexible neural electrodes based on polyimide tape and gold sheet

**DOI:** 10.1007/s13534-023-00345-0

**Published:** 2024-01-31

**Authors:** Hyunbeen Jeong, Taekyung Lee, Jisung Kim, Hee Soo Jeong, Sang Beom Jun, Jong-Mo Seo

**Affiliations:** 1 Electrical and Computer Engineering, Seoul National University, 1, Gwanak-ro, Gwanak-gu, Seoul, 08826 Republic of Korea; 2 Inter-university Semiconductor Research Center (ISRC), Seoul National University, 1, Gwanak-ro, Gwanak-gu, Seoul, 08826 Republic of Korea; 3 Institute of Engineering Research at Seoul National University, Seoul National University, 1, Gwanak-ro, Gwanak-gu, Seoul, 08826 Republic of Korea; 4 Graduate Program in Smart Factory, Ewha Womans University, 52, Ewhayeodae-gil, Seodaemun-gu, Seoul, 03760 Republic of Korea; 5 Department of Electronic and Electrical Engineering, Ewha Womans University, 52, Ewhayeodae-gil, Seodaemun-gu, Seoul, 03760 Republic of Korea; 6 Department of Brain and Cognitive Sciences, Ewha Womans University, 52, Ewhayeodae-gil, Seodaemun-gu, Seoul, 03760 Republic of Korea; 7 Biomedical Research Institute, Seoul National University Hospital, 101, Daehak-ro, Jongno-gu, Seoul, 03080 Republic of Korea

**Keywords:** Polyimide tape, Gold sheet, Neural electrodes, UV laser

## Abstract

This research was conducted to apply polyimide tape, which has the advantages of low price ans strong adhesive strength, to the neural electrode process. In addition, to maximize the low-cost characteristics, a fabrication process based on UV laser patterning rather than a photolithography process was introduced. The fabrication process started by attaching the gold sheet on the conductive double-sided tape without being torn or crushed. Then, the gold sheet and the double-sided tape were patterned together using UV laser. The patterned layer was transferred to the single-side polyimide tape. For insulation layer, electrode site opened single-sided polyimide tape was prepared. Polydimethylsiloxane was used as an adhesion layer, and alignment between electrode sites and opening sites was processed manually. The minimum line width achieved through the proposed fabrication process was approximately 100 $\mu$m, and the sheet resistance of the conductive layer was 0.635 $\Omega$/sq. Measured cathodal charge storage capacity was 0.72 mC/cm^2^ and impedance at 1 kHz was 4.07 k$\Omega$/cm^2^. Validation of fabricated electrode was confirmed by conducting 30 days accelerated soak test, flexibility test, adhesion test and ex vivo stimulation test. The novel flexible neural electrodes based on single-sided polyimide tape and UV laser patterned gold sheet was fabricated successfully. Conventional neural electrode fabrication processes based on polyimide substrate has a disadvantages such as long fabrication time, expensive costs, and probability of delamination between layers. However, the novel fabrication process which we introduced can overcome many shortcomings of existing processes, and offers great advantages such as simplicity of fabrication, inexpensiveness, flexibility and long-term reliability.

## Introduction

In recent decades, various neural prosthetic devices have been developed to treat neurological diseases. Representative examples include cochlear implants for hearing restoration, deep brain stimulation for the treatment of brain disorders, cardiac implants for patients with electrical problems of heart, and retinal prostheses to restore the vision field [1–6]. To develop such a neural prosthetic device, it is important to design nerve electrodes that transmit electrical signals to the desired nerve cells. Furthermore, for the stable operation of the neural electrodes *in vivo* environment, an appropriate material should be used as a substrate. A variety of materials, including metal, silicon, glass, and polymer have been used in neural prosthetic devices, and polymers have recently received the most attention due to their flexibility, lightness, and ease of processing [7–9].

The first decision to make when designing a polymer based electrode is which polymer to use as the substrate. To achieve this, various factors that can affect the properties of neural electrodes, such as cytotoxicity, water absorption rate, density, Young’s modulus, and permeability must be considered. Past research has shown that polymers such as polyimide, parylene-C, and polydimethylsiloxane (PDMS) are biocompatible substrates for neural electrodes [10–14].

Among them, polyimide is the most widely used material for neural electrodes due to its negligible cytotoxicity and an appropriate level of Young’s modulus and density. Moreover, a thin polyimide multilayer film can be manufactured using spin coating, making it easy to achieve a desired thickness or apply existing MEMS (micro-electro-mechanical systems) processes [15, 16]. Due to these advantages, various neural electrodes using polyimide as a substrate have been manufactured.

However, delamination between metal-polyimide or polyimide-polyimide is a major disadvantage of polyimide-based neural electrodes, which occurs due to the relatively high water absorption rate [17–19]. It is a serious problem that affects the long-term reliability due to a significant decrease in the adhesion of the interface over time in an *in vivo* environment. In order to overcome this, various complementary methods have been proposed, such as controlling the curing temperature of the polyimide layer, increasing adhesion by forming a silicon carbide or titanium layer at the electrode area, or preventing delamination between layers due to moisture absorption. [20–23]. However, these methods make the fabrication process much more complex and increase manufacturing time and cost.

To overcome the delamination and complex fabrication of the existing polyimide-based neural electrodes, in the previous paper, the authors reported the fabrication process with laser patterned double-sided tape and gold sheet for the fabrication of the neural electrodes [24]. However, the uniformity and the stability of manufactured results were not at a satisfying level and proper level of validation had not proceeded. In this study, to overcome the shortcomings of the traditional polyimide-based neural electrode fabrication process and our previous research, a new polyimide tape-based process is proposed. Although several researchers have fabricated polyimide tape-based sensors in the past, the fabrication and verification of neural electrodes array are the first of their kind [25, 26]. Furthermore, the proposed process can fabricate neural electrodes much faster and cheaper than existing processes, while significantly suppressing delamination at the interface between layers. The electrode fabricated in this way can be used as a nerve stimulating electrode at various locations in the form of planner. Also it is expected that if an appropriate anchoring method is introduced, it can be used as a cuff electrode by wrapping the nerve such as vagus nerve or sciatic nerve due to its high flexibility.

In section 2, the materials and the equipment used in our fabrication process were introduced. Also, the detail of the updated fabrication process was described. The whole process of the fabrication process was introduced step by step. In section 3, the evaluation of the suggested fabrication process was presented. First of all, to estimate the performances of the fabrication process, minimum line width, line resistance, and sheet resistance were measured. Second, to evaluate the performance of fabricated electrodes, cathodal charge storage capacity (CSC_c_) and the impedance of fabricated electrodes were represented. Next, to show the durability and the resistance to the delamination of the electrodes, a 30-days accelerated soak test, flexibility test, and adhesion test proceeded. The adhesion test of the two interfaces, metal-polyimide and polyimide-polyimide was conducted respectively. Last, to confirm the feasibility of fabricated electrodes, *ex-vivo* stimulation test was conducted.

## Materials and methods

### Materials

**Fig. 1:**
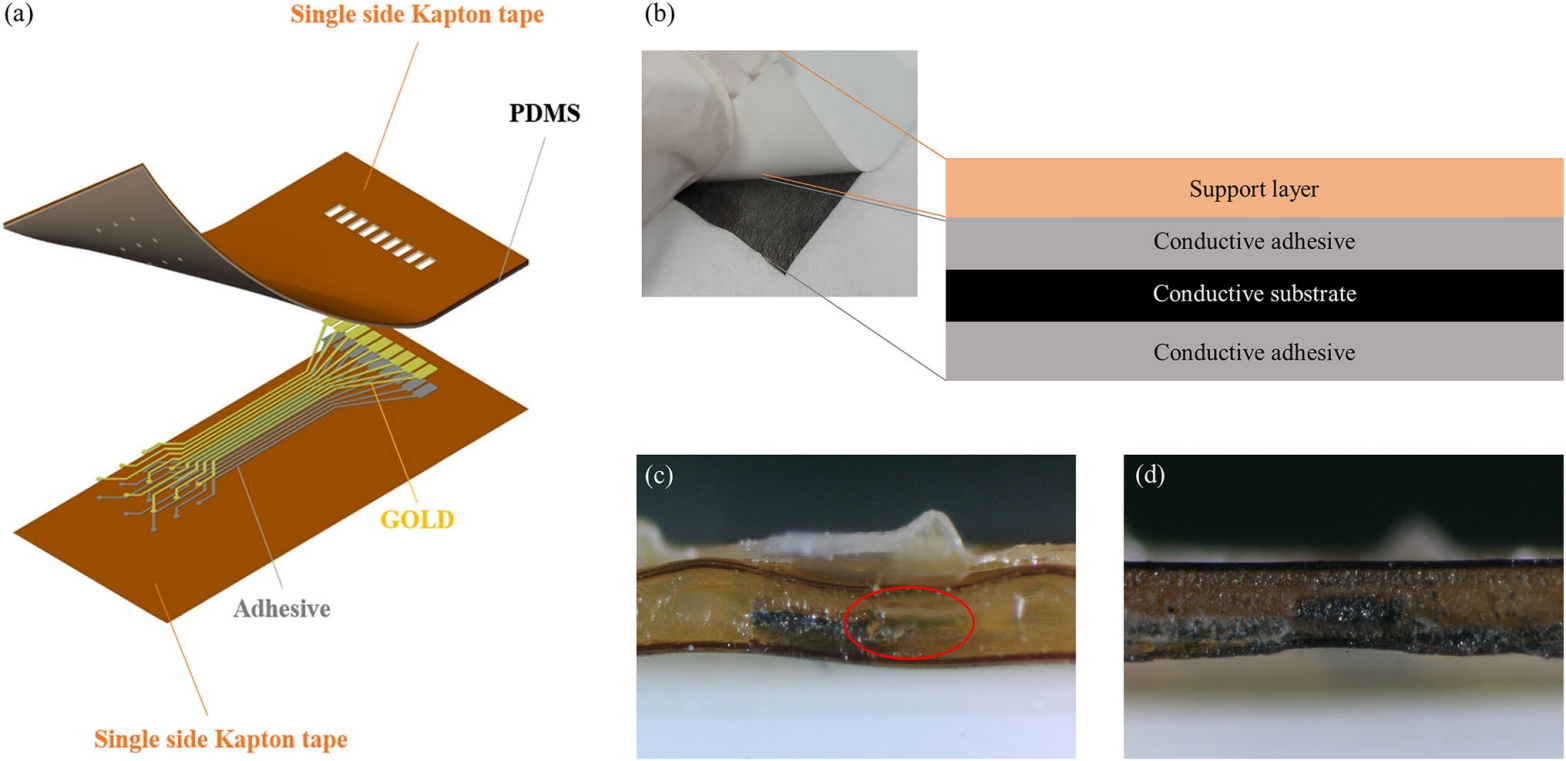
Overview of fabricated electrodes. **a** Basic structure of flexible neural electrodes. **b** Structure of double-sided tape. Double-sided tape is composed of four layers. Support layer is the white sheet in the left picture. Two conductive adhesive layers and a conductive substrate consisted of one adhesion layer which represented as black sheet in the left picture. Those conductive layers were represented as an ‘Conductive tape’ in the Figs. 2 and 3. **c** Cross-section view of electrode fabricated by direct insulation **d** Cross section view of electrode fabricated by PDMS assisted integration

Materials that are widely used and easy to get were selected to fabricate flexible neural electrodes. Double-sided tape with the conductive acrylic adhesive (YCNW50D, YoungJin Co, South Korea), Single-sided polyimide tape (Alphaflon Co, South Korea), 1 $\mu$m thickness gold sheet (DongYang Gold Silver Leaf & Powder Industry, South Korea) with 99.9 % purity and the Polydimethylsiloxane (PDMS, Dow Inc, USA) were used as materials. The structure of double-sided tape was introduced in Fig. 1b. The conductive substrate of double-sided tape is polyester with thin Ni-Cu film plated on both sides. The thickness of the substrate is 30 $\mu$m and the thickness of the acrylic adhesive is 10 $\mu$m for both sides. Single-sided polyimide tape consists of 25 $\mu$m thickness polyimide and 40 $\mu$m thickness silicone adhesives. For manufacturing, UV laser (Marcs laser Inc, South Korea), Spin coater (Dong-Ah trade Co, South Korea), O_2_ plasma treatment system (JP materials, South Korea), Centrifuge (Eppendorf, Germany), and the muffle furnace (SH SCIENTIFIC, South Korea) were used.

### Fabrication process

**Fig. 2:**
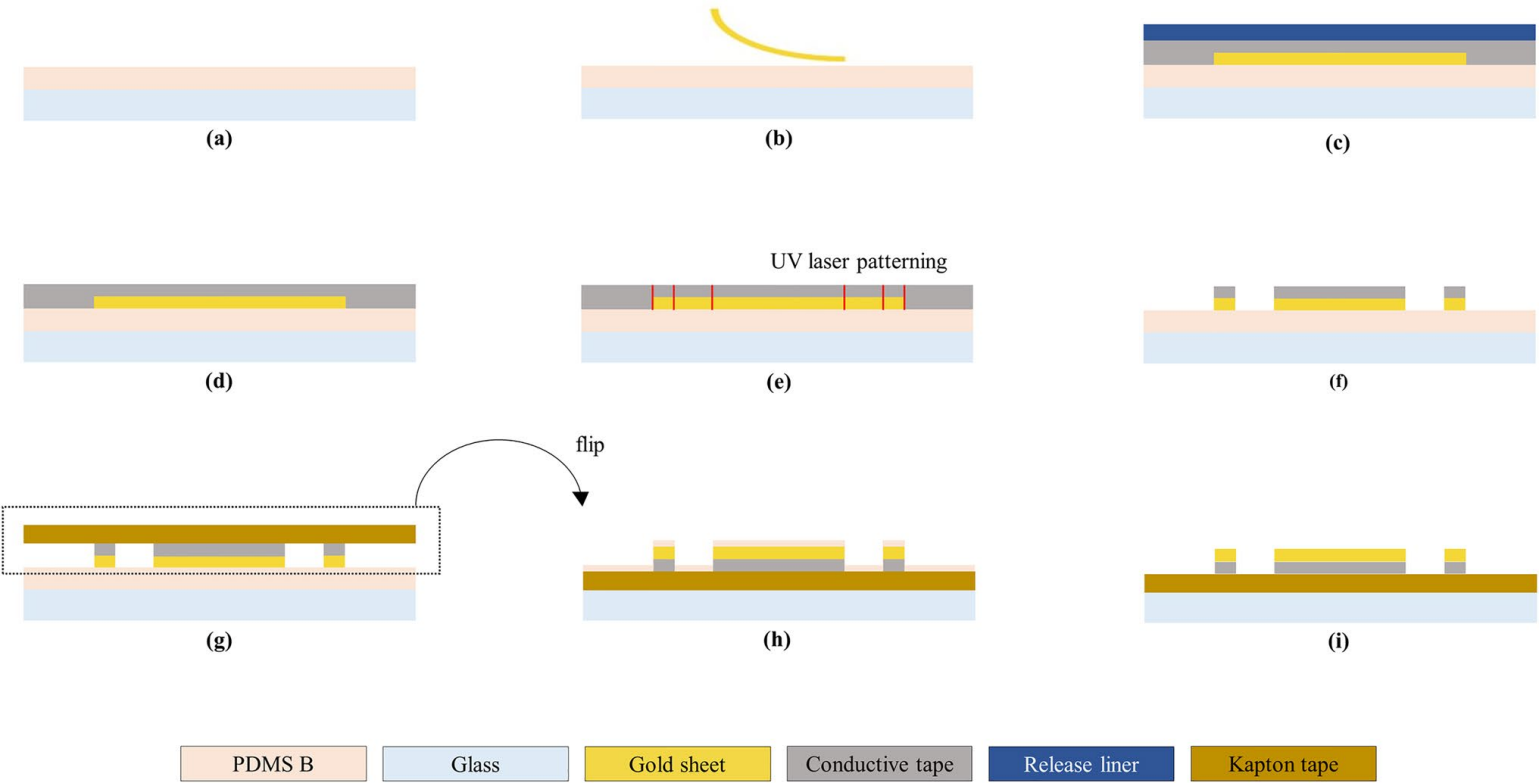
Overview of electrode layer fabrication process. **a** Coat slide glass with curing agent of PDMS using spin-coater (500rpm for 30 s). **b** Place the gold sheet gently on top to make it even. **c** Attach double—sided conductive tape on the gold sheet. **d** Remove the release liner of the conductive tape. **e** Pattern the conductive tape—gold sheet layer using UV laser. **f** Remove the residual area manually. **g** Attach single side polyimide tape on the pattern. **h** Flip the whole fabricated structure and fix on the glass slide with tape. **i** Rinse with acetone to remove remaining PDMS curing agent. This becomes the electrode layer

**Fig. 3:**
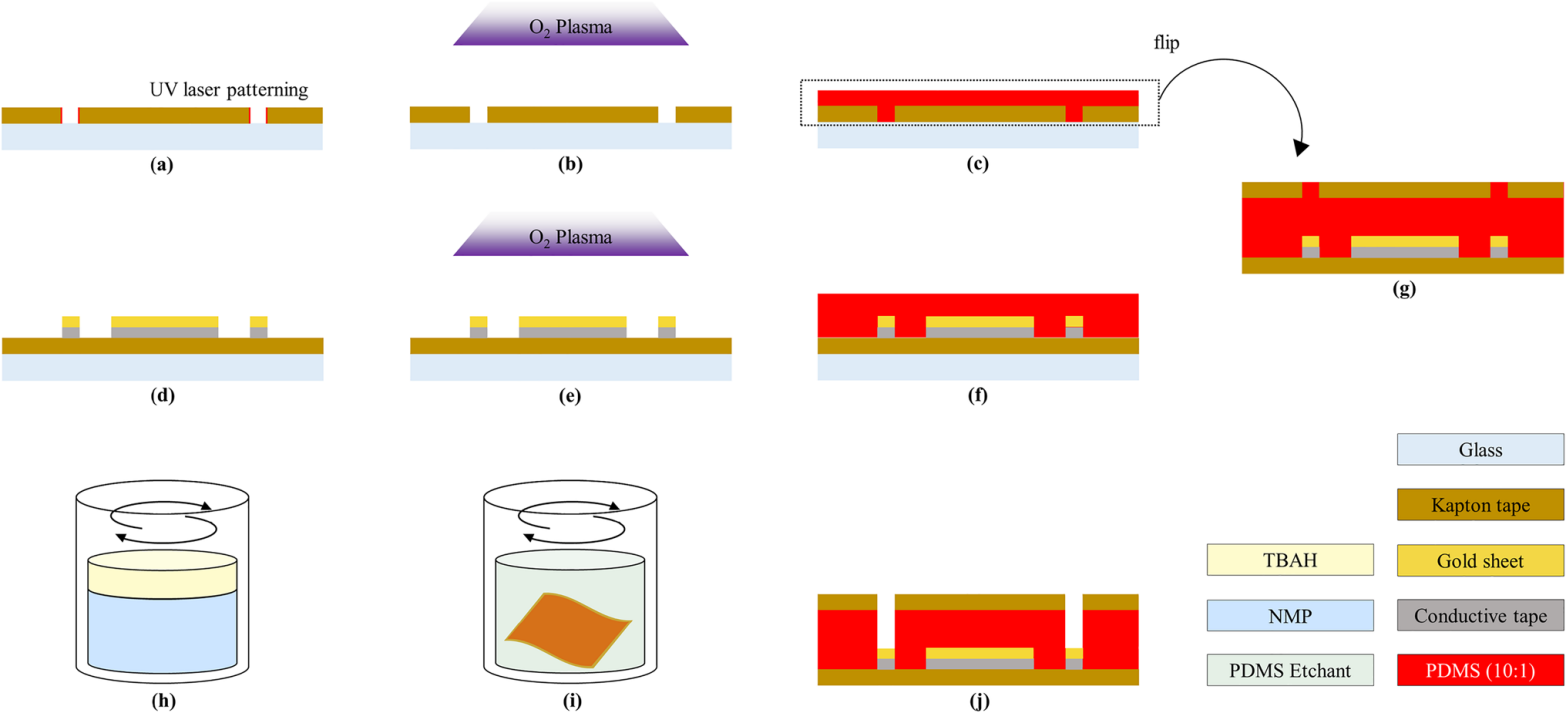
Overview of insulation layer fabrication process. **a** Fix the single sided polyimide tape on the glass slide and pattern the opening site on the polyimide tape using a UV laser. This becomes the insulation layer. **b** To achieve better adhesion between the polyimide tape and the PDMS, O_2_ plasma surface treatment was processed. **c** Spin coat the PDMS on the site opened insulation layer. **d** Prepare the electrode layer manufactured according to Fig. 2. **e** Conduct O_2_ plasma surface treatment on electrode layer as in (**b**). **f** Spin coat the PDMS on the electrode layer. **g** Slightly combine the PDMS coated face of the electrode layer and the insulation layer to avoid bubbles. **h** Prepare PDMS etchant by mixing NMP and TBAF in a 3:1 ratio. **i** Put (**g**) to (**h**) and stirr at 70 rpm for 20 min to etch the PDMS on electrode sites. **j** Cross section view of fabricated flexible neural electrodes

The basic structure of flexible neural electrodes was represented in Fig. 1a. The whole fabrication process was introduced in Figs. 2 and 3. The overall fabrication time was less than 30 min except curing time of PDMS and the material cost was less than a few dollars.

#### Preparation of electrode layer

To fabricate flexible neural electrodes with the gold sheet and the double-sided tape, the first priority was to layer the gold sheet and conductive tape uniformly. Due to the very thin and fragile nature of the gold sheet, it was necessary to establish an appropriate adhesion method to attach it without breaking or lifting. Because the van der Waals force between the gold sheet and the tape unintentionally causes the two layers to stick together, the gold sheet must be firmly fixed before attaching with the tape. Therefore, the appropriate adhesion layer was needed and after testing several bio-compatible resins and curing agents, polydimethylsiloxane curing agent (Sylgard 184, silicone elastomer curing agent) was selected as the material for fixing the gold sheet. This worked well as an adhesion layer by firmly fixing the gold sheet to the glass slide without breaking it. The adhesion process was conducted as follows: First, the curing agent of PDMS was coated on the glass slide using spin coater. Since sufficient thickness should be ensured to provide appropriate adhesion, spin coating was performed at 500 rpm for 30 s. Next, the gold sheet was placed gently on top of the adhesion layer. The double-sided conductive tape was attached on the gold sheet evenly. Finally, by removing the release liner of the tape, preparation for laser patterning was ended.

#### Patterning and transfer

In this fabrication process, a UV laser was used for the electrode patterning. During the laser patterning process, over irradiation may cause problems such as pattern distortion or burning of the pattern edge due to physical damage. To prevent this problems, the condition that could transmit the minimum power to the conductive layer while cutting off at once was found. The patterning conditions were 5 W output power, 22 mm/s laser speed, 30 kHz output frequency, and 10 $\mu$s pulse width. After patterning, residue parts should be removed carefully. Single-sided polyimide tape was used to transfer the patterned layer without any distortion. Using the adhesive side of the tape, the gold layer of the pattern was revealed. Since some curing agent of PDMS may remain on the surface of the pattern, cleaning was performed with acetone for 10 s.

#### Preparation of insulation layer

To open the electrode sites in the insulation layer, they were patterned using the UV laser as Sect. 2.2.2. The pattern of the electrode sites was designed slightly larger than the metal pattern to prevent the overflowing of uncured PDMS over the electrode sites, which will be mentioned later. To clean up the polluted area after patterning, the insulation layer was rinsed in the acetone for 20 s.

#### Integration for insulation

The electrode layer and the insulation layer should be integrated well to provide uniform sealing. In the initial trial, single-sided polyimide tape was used as an insulation layer directly. However, since both thicknesses of the pattern and the single-sided polyimide tape were similar, precise and uniform sealing with direct packaging was almost impossible. Figure 1c showed the cross-section image of direct integration. The packaged result was not uniform, and a large air bubble existed near the pattern (red circle). This may cause low reliability, so to overcome this issue, the gap between the electrode layer and the insulation layer was filled with the PDMS as shown in Fig. 1d. 10: 1 ratio of the PDMS resin and the PDMS curing agent were mixed uniformly using a centrifuge for 60 s with 2000 rpm. O_2_ plasma treatment at the laboratory level was conducted to increase the adhesion between the polyimide tape and the PDMS as showun in Fig. 3b and e. The conditions of O_2_ plasma treatment were 150W power, 13.56 MHz frequency, 180 s, and 20 sccm gas flow rate. The uniformly mixed PDMS was spin-coated on the electrode layer and the insulation layer at 2000 rpm for 30 s and 4000 rpm for 30 s, respectively. Those conditions were selected to provide sufficient thickness to cover the pattern. The two layers coated with PDMS was manually aligned and attached very carefully to prevent the air bubbles from getting trapped between the two layers. Lastly, the curing process was proceeded for 4 h, at 80 ^∘^C.

#### PDMS etching for site opening

**Fig. 4:**
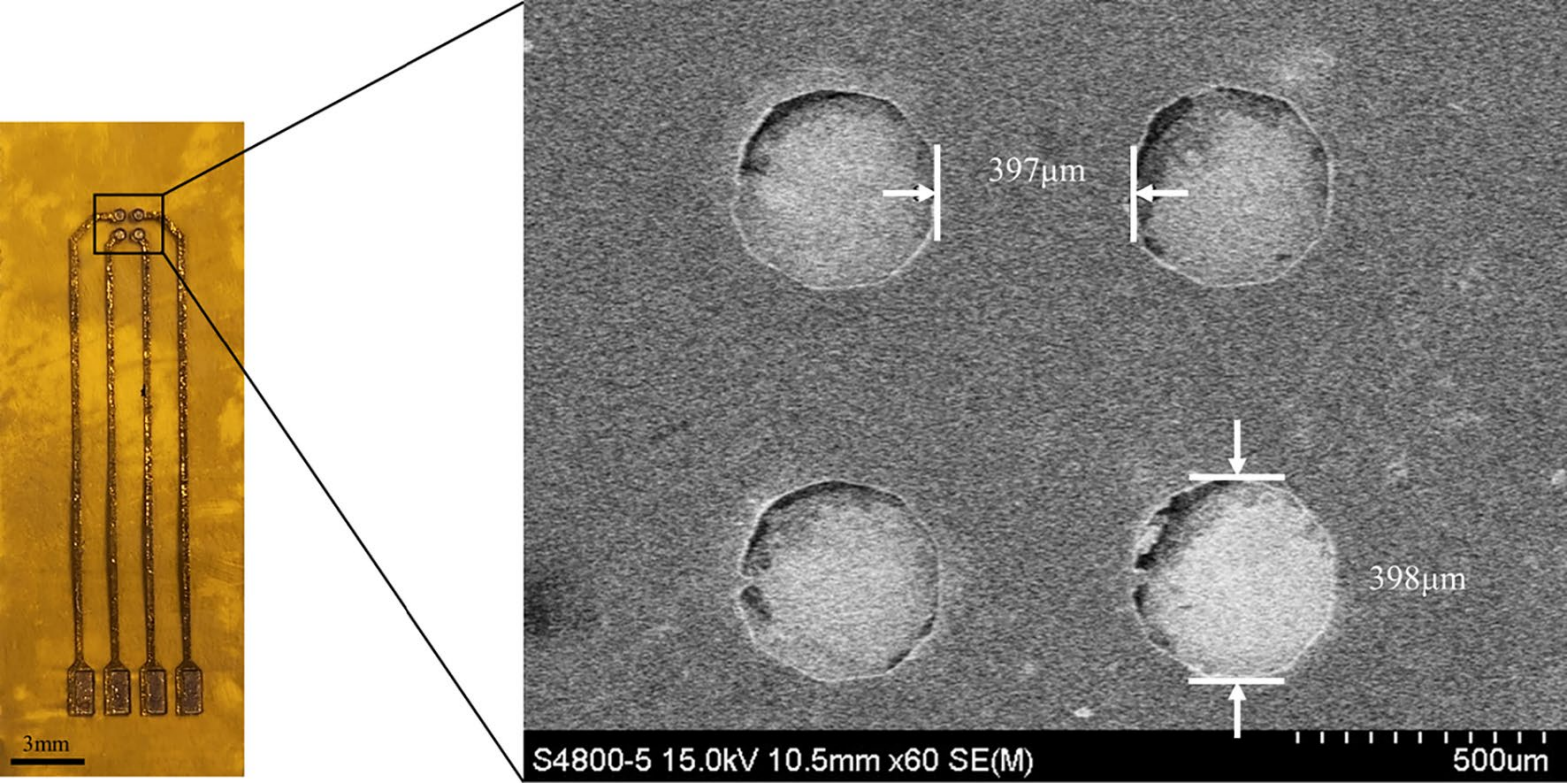
Top-view of the fabricated electrode and the SEM image of the site-opened electrode after PDMS etching process

PDMS etching solution for site opening was prepared by mixing Tetrabutylammonium fluoride solution (TBAF solution, Sigma-Aldrich, USA) in N-methyl-2-pyrrolidinone (NMP, Sigma-Aldrich, USA) by 1:3 ratio. The fabricated sample which was made in Sect. 2.2.4 was immersed into the etching solution and stirred at 70 rpm for 20 min. After etching process, the site-opened electrode was rinsed with deionized water. The fabricated results were shown in Fig. 4.

## Results and discussion

In this section, the fabricated electrode is evaluated from various perspectives. First of all, to check out the distortion during the laser irradiation, the line widths of the pattern after laser patterning were measured. Also, the minimum line width achieved through this fabrication process was confirmed. To prove the performance, sheet resistance of the conductive layer and the line resistance after fabrication were gauged. In addition, cathodic charge storage capacity and impedance of fabricated electrodes were measured. In the case of impedance, measured results right after fabrication and 30 days after being stored in the 75 ^∘^C phosphate-buffered saline (PBS) solution was compared. To show the reliability and the durability of the fabrication process, the accelerated soak test for 30 days and the flexibility test were conducted. Resistance to delamination between the layers was presented using tape test and blister test. Lastly, to make sure it works properly as a neural electrode, *ex vivo* stimulation test were conducted.

### Line width of the pattern

In our suggested fabrication process, patterning was processed using the UV laser. Since the laser is irradiated in a circled area, rather than a dot, it induced the distortion between the intended laser pattern and the actual pattern. Therefore, differences between the expected pattern and the actual pattern after laser patterning should be measured.

In order to examine how much the laser distorts the intended pattern, the line width from 200$\mu$m to 500$\mu$m was patternes at intervals of 50 $\mu$m, and this was measured using a scanning electron microscope (SEM, HITACHI S-4800). The measurement was conducted 10 time respectively for each line width, and the averaged data were shown in Table 1. As we can see in the table, the difference between actual line width and intended line width was about 60 $\mu$m. Since laser engrave both sides of the patterned line, each side was peeled off about 30 $\mu$m. In addition, it was confirmed that the minimum line width was about 100 $\mu$m. Line widths less than this can also be obtained through laser patterning, but in this case, there is a high possibility of breakage during the transfer process. For convenience, the pattern width spoken from the next paragraph is the width of the expected pattern.

**Table 1:** Width comparison between intended line and actual line

Intended line width ($\mu$m)	Actual line width ($\mu$m)
200	123.2 (± 5.67)
250	179.4 (± 5.43)
300	224.4 (± 5.08)
350	263.2 (± 5.74)
400	333 (± 5.22)
450	380.2 (± 5.11)
500	431.4 (± 4.27)

### Line resistance and sheet resistance

The sheet resistance of the conductive layer (multi-layer with the gold sheet and the conductive adhesive) was measured using 4-point probe (FFP-5000, Changmin Inc. South Korea). Measured sheet resistance was 0.635 $\Omega$/sq and the uniformity was about 98 $\%$. Also, line resistance after packaging was measured varying the line width from 200 $\mu$m to 500 $\mu$m with 100 $\mu$m steps. Measurement proceeded using a multimeter and the results of measurements were exploited in Table 2. Each line width was measured 10 times. As shown in the table, the smaller the line width causes the greater variation in resistance. In addition, it was also confirmed that the resistance value was inversely proportional to the line width according to Ohm’s law.

**Table 2:** Line resistance versus line width

Line width ($\mu$m)	Line resistance ($\Omega$)
200	2.00 (± 0.29)
300	1.31 (± 0.40)
400	0.84 (± 0.03)
300	0.75 (± 0.10)

### Charge storage capacity and impedance

**Fig. 5:**
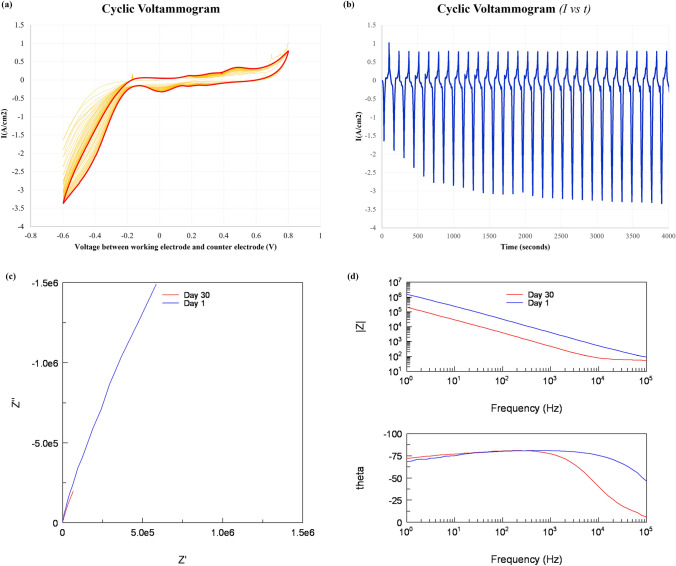
Cyclic voltammogram and normalized impedances of fabricated electrodes. **a** E(voltage)—I(current) curve **b** T(time)—I(current) graph **c** nyquist plot **d** magnitude and phase of impedance

The cathodic charge storage capacity and the impedance of fabricated electrodes after whole fabrication process were measured using impedance analyzer (Solatron SI 1260, AMETEK, USA) and electrochemical interface analyzer (Solatron SI 1287, AMETEK, USA). Cyclic voltammograms with 30 cycles, 20 mV/s scan rate were shown in the Fig. 5. In the Fig. 5a, red line represented the cyclic voltammogram of the last cycle. Figure 5b shows the current versus time graph. The CSC_c_ calculated from the last cycle of the cyclic voltammogram, which is represented with the red line was 0.72 mC/cm^2^. This value was slightly larger than other gold electrodes with hermetic sealing. The CSC_c_ of gold electrode fabricated on cyclic olefin copolymer (COC) substrate was 0.31 mC/cm^2^ and on the liquid crystal polymer (LCP) was 0.32 mC/cm^2^ [27, 28].

Impedance measurement was proceeded from 100 kHz to 1 Hz, 10 mV amplitude. Measured impedance at 1 kHz after fabrication was 4.07 k$\Omega$/cm^2^ magnitude and $-81^\circ$ phase. The normalized results were described in the Fig. 5c and d. Also, after putting electrodes in a PBS solution of 75 ^∘^C for 30 days, impedance of same electrode was measured again. Measured impedance at 1 kHz after 30 days were 0.470 k$\Omega$/cm2 magnitude and $-77^\circ$ phase. Those results were better than the other gold electrodes with hermetic sealing [27, 28]. The superiority of CSC_c_ and impedance characteristics compared to the conventional fabrication processes using metal deposition comes from the high roughness of gold sheet. Compared to deposited gold, the relatively low flatness of the gold sheet and the adhesive had the effect of increasing the effective surface area. This increase made the CSC_c_ and impedance characteristics better.

**Fig. 6:**
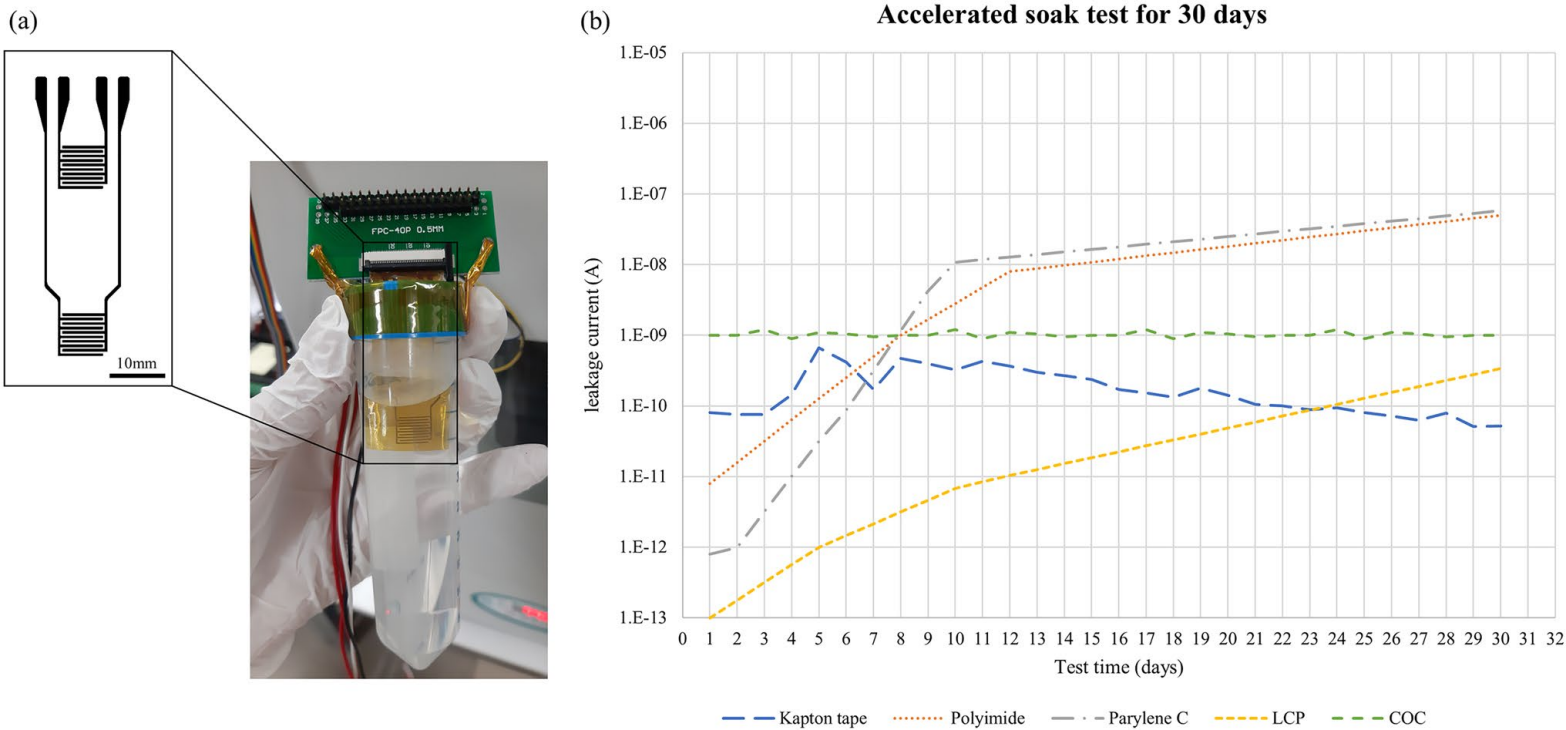
**a** The interdigitated electrode for accelerated soak test immersed in PBS solution **b** The results of accelerated soak test for 30 days. The blue line named ‘polyimide tape’ is the results of our fabrication process. The approximate leakage currents of similar accelerated soak test for polyimide, parylene-C, liquid crystal polymer (LCP), cyclic olefin copolymer (COC) were plot together [15, 25]

### Accelerated soak test

To estimate the long-term reliability of our fabrication process, 30-days accelerated soak test was conducted. Two interdigitated electrode patterns (IDE) with line width and line spacing of 400 $\mu$m were vertically arranged, and only one was immersed in phosphate-buffered saline solution (PBS) for use as a target. The other one was used as a reference without being immersed. The temperature of the PBS was maintained at 75 ^∘^C, and the applied voltage was 5V. Measurement was proceeded using a data acquisition multi-meter system (DAQ6510, Keithley, USA) and picoammeter (6485 picoammeter, Keithley, USA) for 30 days. For the reliability of the results, the experiment was conducted five times and the averaged result was shown in Fig. 6 with standard deviation. We also showed a rough estimate of IDE soak test results based on different polymer substrates reported in previous studies in the same figure [15, 25] for comparison. Each experiment has a different starting point because the equipment and settings used in each experiment are different. However, comparing the tendency of each experimental results, the water absorption rate and long-term reliability of each polymer substrate could be analyzed. Our results were almost similar with cyclic olefin copolymer (COC), which is known to provide hermetical sealing and the results were taken by the same experiment environments. Also, the leakage current value after 30 days was almost the same as the liquid crystal polymer (LCP) which is known as providing hermetic sealing and was much better than parylene-C or polyimide, which has a significantly poor water absorption rate. By applying the 10-degree rule, 30 days in 75 ^∘^C could be roughly estimated to 480 days in human body temperature (37 ^∘^C).

We were able to demonstrate two facts from this experiment: the low moisture permeability of polyimide tape and the strong adhesion between PDMS and polyimide tape. Even though PDMS of several tens of micro-thickness has almost no resistance to moisture, polyimide tape, which has high moisture resistance prevented the leakage current from increasing during the test period. In addition, when PDMS is adhered to a polymer substance such as parylene-C and exposed to an *in vitro* environment, the interface is usually broken within a short period of time. However, the bond between the interfaces did not break throughout this experiment. This means that the bond between the PDMS and the polyimide tape is strong, and the blister test was conducted in Sect. 3.6 to confirm the adhesion strength between the interfaces.

### Flexibility test

**Fig. 7:**
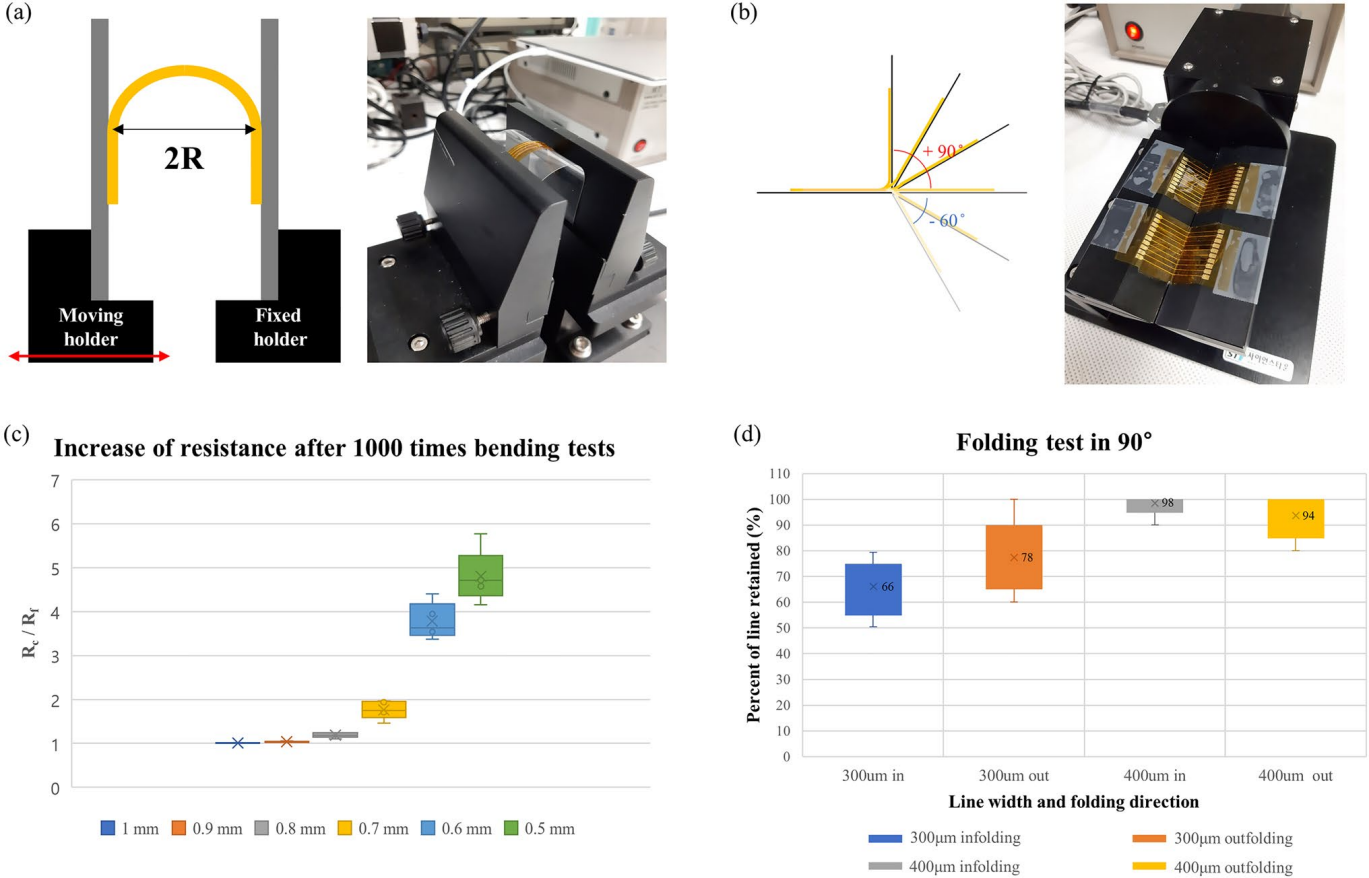
Experiment image and results of flexibility test **a** R-bending test **b** folding test **c** The results of bending tests **d** The results of folding test at 90 degrees

To verify the reliability of our fabricated results in the flexible application, two kinds of flexibility tests were performed. First, to represent the resistance variance during bending, the R-bending test was conducted using a 1-axis motion controller (STM-1-TS, ST1 corp. South Korea) in Fig.7a. Second, to check out the failure in a folding environment, the folding test proceeded using a 1-axis motion controller (STM-1-USB, STI corp, South Korea) in Fig.7b.

For R-bending test, the radius of bending (R) was varied from 5 mm to 10 mm with 1 mm step and the line width was 500 $\mu$m. For each radius, resistance was checked every 100 cycles, and measurements were conducted up to 1000 cycles. The speed of bending test was 100 mm/s. The ratio between the measured resistance of the first bending (Rf) and the measured resistance of the bending at every 100 cycles (Rc) were represented in Fig. 7c. No electrical failure was founded during the measurement and the increase of resistance was in the reasonable range. However, the increase in resistance did not return to its original state even if measured in a planar state after finishing the test. The reason for those increases was due to the non-uniformity of the PDMS layer while attaching the conductive layer to the substrate. Due to the imperfections during fabrication, the fabricated result is uneven in thickness. So, bending stress was collected at the thinnest point during bending, causing damage in that part. Those imperfections should be improved in future work.

For folding tests, the folding angle was set to 90 degrees in in-folding and 90 degrees in out-folding, and a total of 10,000 folding was performed. 30 mm long 10 line patterns with line width of 300 $\mu$m and 400 $\mu$m respectively, were prepared. The standard for electrical failure of line resistance was set over 100 $\Omega$ so a resistance greater than 100 $\Omega$ after folding was considered to be broken. For each degrees and line width, the experiment was conducted for 5 times respectively, and the average results were shown in Fig 7d. If the line width is thin, which is 300 $\mu$m, as shown in figure, the possibility of electrical failure increases. Also, there was a slightly higher probability of failure in the in-folding situation. This difference in probability of failure according to the folding angle seems to be because the conductive layer is not placed on the neutral plane. If the conductive layer can be accurately located on the neutral plane by improvement of fabrication process, this difference of failure probability seems reducible. When the line width was large at 400 $\mu$m, 10,000 times of folding did not significantly affect the electrical connection of the electrode.

### Adhesion test

To show that the fabricated electrode is resistant to inter-layer delamination, it is necessary to confirm that the adhesive force between each layer is sufficient. To check out this, two tests were conducted. First, a tape test was performed to examine the adhesion between the conductive layer (combination of gold sheet and double-sided tape) and the polyimide tape. Next, a blister test was conducted to confirm that the adhesion between the substrate and packaging layer was large enough. For the tape test, a 1 cm square size conductive layer put on the polyimide tape was prepared. To show that the bonding was maintained in the in-vitro environment, the prepared sample was immersed in PBS solution at 75 ^∘^C for 30 days. The tape test proceeded with scotch tape. (3 M, USA) During the tape test, no delamination or peeled-off particles were found. From this, it was confirmed that the adhesion between the conductive layer and the substrate was strong enough to withstand the internal body environment. For blister test, the sample with 3 mm size blister, the holder with 2 mm hole, and N_2_ gas injector with pressure controller were prepared. The blister of the sample was aligned to the center of the hole and the sample was fixed with instant glue (Loctite SG Easy Brush, Loctite, Germany). Then, the pressure applied to the blister was gradually increased until the destruction of bonding between layers was observed. Also, to demonstrate the delamination of packaging does not occur in the in-vitro environment, the samples placed in the PBS solution at 75 ^∘^C for 30 days were tested. All samples showed the results that the instant glue layer and sample detached before the delamination between PDMS-polyimide tape occurred. The detachments between the instant glue and the samples occurred when a pressure of about 150 psi was applied. Through these results, it can be seen that the critical pressure at which adhesion between PDMS-polyimide tapes is destroyed was greater than 150 psi even after 4 weeks of exposure to a 75 ^∘^C PBS solution. The corresponding value is significantly higher than 130 psi, which is the critical pressure of the polyimide-polyimide interface in the same environment [19]. Therefore, it was confirmed that the interfacial adhesion was significantly improved through our suggested process.

### *Ex-vivo* stimulation test

**Fig. 8:**
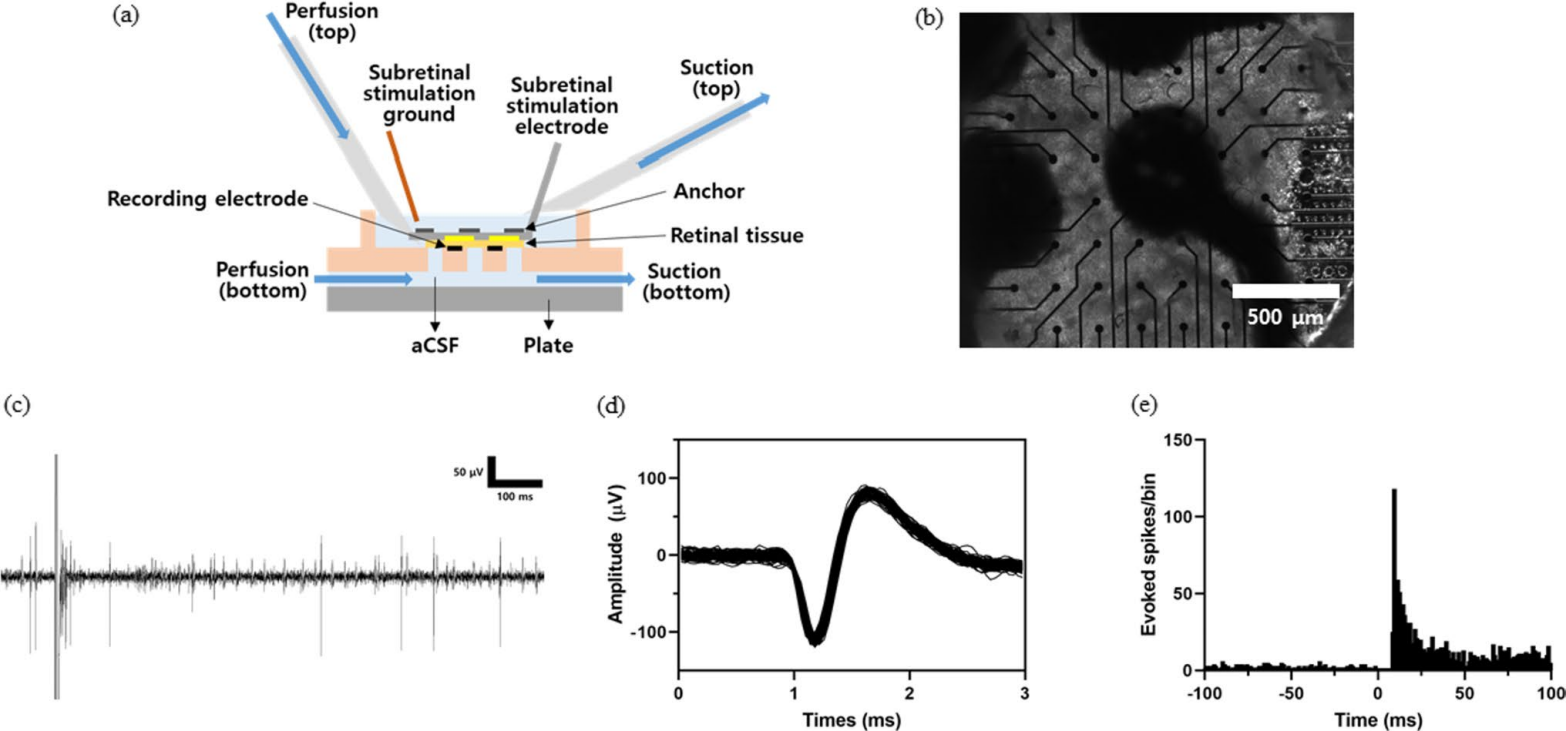
Experiment setup and results of *ex-vivo* stimulation test **a** schematic diagram of the setup **b** a large area electrode located in the middle indicates the electrode applied with electrical stimulation **c** evoked retinal ganglion cell (RGC) responses to a 3 V voltage pulse applied by one stimulating micro-electrode **d** Sorted spike waveform of single unit **e** post-stimulus time histogram (PSTH) of electrically evoked RGC responses

To confirm the feasibility of the fabricated electrode, an ex-vivo stimulation test was conducted. The retina of mouse (C57BL/6N, male, 8 weeks, KOATECH, Pyeongtaek-si, Korea) was used for the stimulation test. All animal procedures were approved by the Institutional Animal Care and Use Committee (IACUC) at Ewha Womans University (IACUC 22-007). The setup for the ex-vivo test was described in Fig. 8a. Before the test, to harvest the fresh retina from the mouse was anesthetized through inhalation using isoflurane for 5 min. After the cervical dislocation, both eyes were enucleated and the whole layers of retina were harvested. The retinal patch was prepared under dim red light in artificial cerebrospinal fluid (aCSF) (124 mM NaCl, 5 mM KCl, 1.15 mM KH_2_PO_4_, 1.15 mM MgSO_4_, 10 mM Glucose, 25 mM NaHCO_3_, 5 mM CaCl_2_) bubbled with 95% O_2_ and 5% CO_2_ to maintain a pH of 7.3 $\sim$ 7.4 at room temperature. Then, for the neural recording, prepared retinal patch was loaded on the 60 channel recording MEA (perforated microelectrode array; 60pMEA200/30iR-Ti, 30 $\mu$m of electrode site diameter and 200 $\mu$m of spacing; Multi Channel Systems MCS GmbH, Reutlingen, Germany). In order to perform subretinal stimulation, the ganglion cells of the retina were loaded to be positioned downwards. By putting the electrodes on the retina, like Fig. 8b, preparations for ex-vivo stimulation test was finished.

The results of stimulation test were plotted from Fig. 8c–e. For the stimulation, cathodic-first biphasic pulse was generated from stimulus generator (multi channel systems MCS GmbH, Reutlingen, Germany). The voltage stimulation was used and the stimulation parameters were set as follows: Phase amplitude: 3 V, Phase duration: 500 $\mu$s, inter-pulse interval: 999 ms, stimulation frequency: 1 Hz. In Fig. 8c, evoked response of retinal ganglion cell (RGC) after stimulation was described. Because the size of the stimulation electrode is larger than the size of the RGC, a single stimulation activates more than one RGC. Therefore, multiple RGCs respond to a single nerve stimulus, and these responses were spike-sorted using a threshold voltage of -32 $\sim$ -12 $\mu$V, as represented in Fig. 8d. In addition, in Fig. 8e, it is shown in the post-stimulus time histogram (PSTH) that the response of the RGC exhibits a clear contrast before and after the stimulation moment (0 s). This confirms that the fabricated neural electrode can successfully stimulate all the nearby nerves in the site.

## Conclusion

In this study, the fabrication process and validation of flexible neural electrodes using single-sided polyimide tape and gold sheet patterned with UV laser were introduced. The charge storage capacity and the impedance of the fabricated neural electrodes were at an appropriate level, and they showed outstanding performance in the flexibility test. To verify the long-term reliability of the electrode, the accelerated soak test to measure leakage current and the adhesion test to confirm the resistant to delamination were performed, and test results showed excellent reliability. Also, the feasibility of fabricated electrodes was investigated through ex-vivo stimulation test.

The proposed process only required less than 15 min of fabrication time except for the PDMS curing, and the material and process cost were very low. In addition, unlike the conventional MEMS process, the use of substances that can cause environmental pollution was minimized.

The introduced process method has several areas for improvement. First, the minimum line width over 100 $\mu$m should be improved. The minimum line width using direct patterning of UV laser seems to be close to about 50 $\mu$m, but there was considerable difficulty in achieving a minimum line width of 100 $\mu$m or less due to damage in the transfer process. Second, the aligning method that is currently being done by hand must be changed. It is almost impossible to perform alignment at line widths less than 100 $\mu$m by manual alignment. Finally, long term reliability over a longer period of time should be verified in an in vivo environment. These points will be reported in further studies.
